# A Novel Role for Raloxifene Nanomicelles in Management of Castrate Resistant Prostate Cancer

**DOI:** 10.1155/2014/323594

**Published:** 2014-02-06

**Authors:** Sebastien Taurin, Hayley Nehoff, Thalita van Aswegen, Rhonda J. Rosengren, Khaled Greish

**Affiliations:** ^1^Department of Pharmacology and Toxicology, University of Otago, Dunedin, New Zealand; ^2^Department of Oncology, Faculty of Medicine, Suez Canal University, Ismailia, Egypt

## Abstract

Of patients with castrate resistant prostate cancer (CRPC), less than 25–33% survive more than five years. Recent studies have implicated estrogen, acting either alone or synergistically with androgens in the development of castrate resistant prostate cancer. Several *in vitro* and *in vivo* studies, as well as a limited number of clinical trials, have highlighted the potential of selective estrogen receptor modulators, such as raloxifene (Ral) for the treatment of castrate resistant prostate cancer. However, the poor oral bioavailability and metabolism of selective estrogen receptor modulators limit their efficiency in clinical application. To overcome these limitations, we have used styrene co-maleic acid (SMA) micelle to encapsulate raloxifene. Compared to free drug, SMA-Ral micelles had 132 and 140% higher cytotoxicity against PC3 and DU 145 prostate cell lines, respectively. SMA-Ral effectively inhibits cell cycle progression, increases apoptosis, and alters the integrity of tumor spheroid models. In addition, the micellar system induced changes in expression and localization of estrogen receptors, epidermal growth factor receptor (EGFR), and downstream effectors associated with cell proliferation and survival. Finally, SMA-Ral treatment decreased migration and invasion of castrate resistant prostate cancer cell lines. In conclusion, SMA-Ral micelles can potentially benefit new strategies for clinical management of castrate resistant prostate cancer.

## 1. Introduction

Prostate cancer is the most common noncutaneous malignant neoplasm and the second leading cause of male cancer-related deaths in Oceania, Europe, and North America [1]. For the 25 to 40% of patients not cured by the initial treatments of prostatectomy or radiation therapy, the cancer inevitably reoccurs and metastasizes to distant organs [1, 2]. The standard treatment for metastatic prostate cancer is surgical or chemical castration which reduces circulating androgens (<50 ng/dL) and suppresses the activity of the androgen receptor (AR) [3]. However, despite an initial 12–18 months of regression, patients frequently relapse and a more aggressive cancer progresses to a castrate resistant status [4]. The 5-year relative survival rate for patients with castrate resistant prostate cancer (CRPC) is approximately 25–33% [5]. The initiation and progression of CRPC are not well understood and may involve multiple mechanisms such as the activation of tyrosine kinase receptors by growth factors, the loss of cell cycle regulators or genetic mutations of the androgen receptor [6]. Therapeutic options for CRPC are limited in their efficacy, as the disease inevitably progresses to metastasis.

Recently, several *in vitro* and preclinical animal studies have involved estrogens alone or synergistically with androgens in the progression of prostate cancer [6–11]. In the clinic, the significance of estrogen plasma levels as a predictor of prostate cancer development remains controversial [12]. Recently it has been demonstrated that prostate tumor growth may rely on systemic circulation levels of steroids and on local steroid production by prostate cancer cells [8, 13, 14]. Multiple isoforms of both estrogen receptor (ER)*α* and ER*β* are differentially expressed in the prostate and contribute to cellular homeostasis. In a disease state, ER*β*1 expression gradually reduces as the cancer progresses towards higher grade. However, ER*β*1 is highly expressed in prostate tumors that have metastasized to the bone and lymph nodes [15]. Less is known about the contributions of the other isoforms ER*β*2–5 [16]. Other studies reported a low ER*α* expression in CRPC and metastatic lesions suggesting a role of ER*α* in tumor development and metastasis [17]. Moreover, the estrogen receptor antagonist, ICI 182, 780, inhibited the growth of the CRPC cell lines DU145 and PC3 cells [18].

In pioneering work in the early 1941s, Huggins and Hodges used diethylstilbestrol (DES), a synthetic estrogen, as a standard therapy for metastatic prostate cancer [19]. Several studies have demonstrated that estrogen receptor modulators can be valuable treatment options and recent preclinical studies have highlighted the use of selective estrogen receptor modulators (SERMs) for the prevention and treatment of CRPC [20]. Using different generations of SERMS (i.e., tamoxifen, raloxifene, or toremifene), several studies have demonstrated the potency of these drugs for the prevention of CRPC *in vitro* and in preclinical studies carried out in rat or mouse models [20–22]. Still, SERMs have shown limited efficacy in clinical trials [23–26]. Raloxifene was approved for the reduction of the risk of invasive breast cancer in postmenopausal women and postmenopausal women with osteoporosis [27], but raloxifene has been also shown to stabilize the progression of prostate cancer in a pilot phase II clinical trial (60 mg/day for 1 year) [25]. These data suggest the potential of raloxifene for the management of CRPC. However, raloxifene's effect is limited *in vivo* by low bioavailability (2%) due to poor solubility, extensive metabolism, and being prone to efflux mechanisms of various transporters such as multidrug resistance-related proteins, or organic anion transporter [28]. Therefore, we have hypothesized that the encapsulation of raloxifene in a nanodelivery platform will improve water solubility, protect the drug from metabolism, and efflux mechanisms and could potentially improve its cytotoxicity against CRPC cell lines.

We have previously developed a nanodelivery platform which exploits the amphiphilic nature of poly(styrene co-maleic acid) (SMA) for the encapsulation of highly hydrophobic drugs [29, 30]. In this study, we synthesized and characterized SMA-raloxifene (SMA-Ral) micelles relative to their drug loading, size, charge, and release rate. We examined the effect of SMA-Ral micelles compared to free raloxifene on cytotoxicity, cell proliferation, and apoptosis in two CRPC cell lines, PC3 and DU145 cells. In addition, we have shown that SMA-Ral alters the integrity of the CRPC tumor spheroids. Finally, we showed that the SMA-Ral inhibits migration and invasion of PC3 CRPC cell line as well as reducing the crosstalk between PC3 and endothelial cells.

## 2. Experimental Section

### 2.1. Materials

Raloxifene hydrochloride (99% purity), cumene terminated poly(styrene-co-maleic anhydride) with an average Mn~1600, N-(3-dimethylaminopropyl)-N-ethylcarbodiimide hydrochloride (EDAC), and sulforhodamine B were obtained from Sigma-Aldrich Ltd.

### 2.2. Methods

#### 2.2.1. Cell Culture

The CRPC cell lines PC3 and DU145 and human umbilical vein endothelial cells (HUVEC) were obtained from American Type Culture Collection (Manassas USA). CRPC cells were maintained in complete growth media DMEM/Ham's F12 supplemented with 5% fetal bovine serum, 2 mM L-glutamine, 100 units/mL penicillin, 100 units/mL of streptomycin, and 2.2 g/L of NaHCO_3_. HUVEC were seeded in complete HUVEC media (EBM-2 basal media containing FBS and growth supplements) as described by the manufacturer (Lonza, Auckland, New Zealand). For all procedures, cells were harvested using TrypLE Express (Life Technologies, Auckland, New Zealand) and were maintained at 37°C in a humidified atmosphere of 5% CO_2_.

#### 2.2.2. Preparation of SMA-Ral Micelles

SMA-Ral micelles were prepared as described previously [30]. Briefly, the hydrolyzed SMA solution was adjusted to pH 5; raloxifene-HCl was dissolved in a minimum volume of DMSO and added to the SMA solution with stirring. EDAC solubilized in distilled water was added to the mixture and allowed to stir for 20 min at pH 5. The solution was then adjusted to pH 11 with 0.1 N NaOH and stirred for 30 min. The pH was readjusted to pH 7.4 with HCl 0.1 N. The clear micelle suspension was ultrafiltered 4 times using a lab-scale ultrafiltration system mounted with a Pellicon XL filter 10 kDa (Merck Millipore, Auckland, New Zealand). The concentrated micelle solution was lyophilized to obtain the final SMA-Ral powder.

#### 2.2.3. Loading of SMA-Ral

A standard curve of raloxifene was prepared in DMSO and measured at 287 nm. Drug content of SMA-Ral was determined by solubilizing SMA-Ral (1 mg/mL) in DMSO and measuring the absorbance at 287 nm in comparison with the standard curve. The loading was expressed as weight % of raloxifene in the final micelle compared to the total weight of recovered SMA-Ral. The SMA-Ral loading was determined as 20%.

#### 2.2.4. Size and Charge of SMA-Ral Micelles

SMA-Ral micelles (4 mg/mL) were solubilized either in NaHCO_3_ (0.1 M, pH 7.4) to determine the size or water to estimate the charge. All measurements for size distribution and zeta potential were carried out using the Malvern ZEN3600 Zetasizer nano series (Malvern Instruments Inc., Westborough, MA). Measurements from three independent experiments were conducted in triplicate.

#### 2.2.5. Drug Release

The release of raloxifene from the micelle construct was evaluated using a dialysis method. SMA-Ral micelles were prepared at a concentration of 1 mg/mL in distilled water. Using a dialysis bag with a 12 kDa molecular weight cutoff, 3 mL was dialyzed against 30 mL of distilled water (pH adjusted to pH 5.5, pH 6.8, or pH 7.4). Over a period of 5 days, 2 mL of sample outside the dialysis bag was removed and the absorbance was measured at 287 nm. The percentage of release was determined by the ratio of the absorbance between the solution outside the bag at defined time points and that within the bag at *t* = 0. All experiments were performed in triplicate. Percentage release is reported as mean ± standard error.

#### 2.2.6. Cytotoxicity of SMA-Ral

PC3 (4 × 10^3^ cells/well) and DU145 cells (1.8 × 10^4^ cells/well) were seeded in 96 well-plates and incubated for 24 h at 37°C in 5% CO_2_ and then treated with 0 to 30 *μ*M concentration range of either free raloxifene or SMA-Ral. The cells were incubated for 72 h and fixed using trichloroacetic acid (TCA). Cell number was determined using the sulforhodamine B assay [31]. The concentration required to decrease cell numbers by 50% (IC_50_) was determined by nonlinear regression using Prism software. The three independent experiments were performed in triplicate.

#### 2.2.7. [^3^H]-Thymidine Incorporation

DNA synthesis in cells was determined using a [^3^H]-thymidine incorporation assay. Briefly, PC3 (20,000 cells/well) and DU145 cells (8 × 10^4^ cells/well) were seeded in 24 well-plates and incubated for 36 h, cells were treated with SMA-Ral or free raloxifene at 2, 5, and 10 *μ*M for 48 h. [^3^H]-thymidine (0.5 *μ*Ci/well) was added for the last 20 h of the treatment. [^3^H]-thymidine incorporation was measured as described previously [32].

#### 2.2.8. Cell Cycle Analysis

PC3 (8 × 10^4^ cells per well) and DU145 (3 × 10^5^ cells per well) cells were seeded in 6-well culture plates in 1.5 mL of complete growth media. Cells were treated with SMA-Ral or free raloxifene at 2, 5, and 10 *μ*M for 48 h. Cell cycle distribution was assessed using propidium iodide staining, as previously described [33]. Samples were analyzed using a FACScalibur flow cytometer (BD Biosciences, San Jose, CA, USA) and the proportion of cells in each of G0/G1-, S- and G2/M-phases were determined using CellQuest Pro software (BD Biosciences, San Jose, CA, USA).

#### 2.2.9. Apoptosis Analysis

PC3 (8 × 10^4^ cells per well) and DU145 (3 × 10^5^ cells per well) cells were seeded in 6-well culture plates in 1.5 mL of complete growth media. Cells were treated with SMA-Ral or free raloxifene at 2, 5 and 10 *μ*M for 48 h. Apoptosis was assessed using Annexin-V-FLUOS/propidium iodide staining, as described previously [33]. Samples were analyzed using a FACScalibur flow cytometer and the proportion of apoptotic cells was determined using CellQuest Pro software.

#### 2.2.10. Western Blot

PC3 cells (4 × 10^4^ cells per well) were seeded in 12-well culture plates in 1 mL of complete growth media and incubated for 36 h. PC3 cells were treated with SMA-Ral or free raloxifene at 2, 5, and 10 *μ*M for 48 h. Cells were lysed in buffer containing 50 mM Tris-HCl (pH 8), 150 mM NaCl, and 1% Triton X-100, 1% SDS, 1 mM NaF, 200 *μ*M sodium orthovanadate, and protease inhibitors (1 *μ*g/mL leupeptin, 1 *μ*g/mL aprotinin, 1 mM PMSF). The lysates were cleared from insoluble material by centrifugation at 20,000 g for 10 min, boiled in Laemmli buffer, subjected to polyacrylamide gel electrophoresis, and analyzed by Western blotting.

#### 2.2.11. Indirect Immunofluorescence Microscopy

Immunofluorescence was performed as described previously [34]. PC3 cells (20,000 cells/well) seeded on glass slides were incubated for 36 h and treated with SMA-Ral or free raloxifene at 5 or 10 *μ*M for 48 h. Cells were washed twice with ice-cold PBS, fixed in 4% paraformaldehyde in PBS for 15 min at room temperature, washed again with PBS, and permeabilized in 0.2% Triton-X100 in PBS for 5 min, followed by incubation with 1% bovine serum albumin in PBS for 1 h. The cells were then incubated with antiestrogen receptor *β* antibody (5 *μ*g/mL in PBS/bovine serum albumin, as above) overnight at 4°C for 1 h and washed four times with PBS, followed by incubation with fluorescein-conjugated goat anti-mouse IgG (10 *μ*g/mL in PBS/bovine serum albumin, as above) for 1 h at room temperature. The slides were washed four more times with PBS, and the coverslips were mounted using Gel/Mount aqueous mounting medium (Fisher, Pittsburgh, PA). The images were taken under a confocal fluorescent microscope.

#### 2.2.12. Cell Migration

Migration of PC3 cells was measured using the *in vitro* cell scratch assay. After cells grown in 6-well plates had reached confluence, a scratch was made with a pipette tip followed by extensive washing with serum-free medium to remove cell debris. SMA-Ral or free raloxifene at 5 and 10 *μ*M or controls (SMA or DMSO) were then added. Cells were allowed to migrate into the scrapped area for up to 20 h at 37°C. At the indicated times, cells were photographed.

#### 2.2.13. Cell Invasion

PC3 cells (4 × 10^4^ cells/mL) were seeded onto growth factor-reduced matrigel invasion chambers (8 *μ*m pore; BD Biosciences) with or without free raloxifene or SMA-Ral 10 *μ*M for 20 h. Lower chambers contained DMEM/Ham's F12 supplemented with the chemoattractant, 5% FBS. Filters were fixed in methanol and stained using Diff Quick staining solutions. Cells from each well were counted under an inverted microscope at 20x magnification. The invasion was expressed as the percentage of cells passing through the matrigel layer over the number of cells counted in the control well without matrigel. Data were collected from three independent experiments, each done in triplicate. Migrated cells were counted, and mean differences (±S.E.) between groups were analyzed using the Student's *t* test.

#### 2.2.14. MMP-9 Activity Assay

PC3 cells were seeded in 6-well plate (1.2 × 10^5^ cells/well) and incubated for 36 h. The cells were washed with PBS and then incubated in serum free media. Cells were treated with either free raloxifene or SMA-Ral 10 *μ*M, DSMO (0.05%) or SMA for 24 h and 48 h. Media was collected, centrifuged to remove cell debris, and freeze dried for 12 h. Samples were rehydrated and mixed with loading buffer (0.4 mol/L Tris, pH 6.8, 5% SDS, 20% glycerol, 0.03% bromophenol blue). Samples were loaded on a 10% SDS-polyacrylamide gel containing 1 mg/mL of gelation. After electrophoresis, the gels were incubated in renaturing solution (2.5% Triton-X-100) for 30 minutes at room temperature and then for 24 h at 37°C in a developing buffer containing 50 mmol/L Tris, pH 7.5, 200 mmol/L NaCl, 4 mmol/L CaCl_2_, and 0.02% NP40. The gels were then stained with Coomassie blue R250, and regions without staining were indicative of gelatin lysis. The gels were briefly rinsed and scanned.

#### 2.2.15. Endothelial Tube Formation Assay and PC3 Coculture

Tube formation was carried out using HUVEC. Briefly, Geltrex (Invitrogen, Auckland, NZ) was allowed to thaw on ice at 4°C overnight. 40 *μ*L was pipetted into a 96-well plate and kept for 30 min at 37°C to allow gelling. HUVECs were seeded in triplicate on the top of Geltrex layer at a density of 1.5 × 10^4^ cells/well. Various concentrations of raloxifene or SMA-Ral (5 and 10 *μ*M) were added into the wells and incubated for 20 h at 37°C in 5% CO_2_ atmosphere. After incubation time, pictures were taken. For the co-culture study. HUVEC (1.5 × 10^4^ cells/well) and PC3 cells (6 × 10^3^ cells/well) were seeded on Geltrex as previously described and treated with free raloxifene or SMA-Ral 10 *μ*M. Following treatments, the cells were incubated for 20 h at 37°C in 5% CO_2_ atmosphere. After incubation time, pictures were taken. The three independent experiments were performed in triplicate.

#### 2.2.16. Tumor Spheroids and Cell Viability via Acid Phosphatase Assay

Tumor spheroids were produced as previously described [35]. Briefly, PC3 cells were trypsinized and the cell suspension (8000 cells) was transferred to a 96-well plate precoated with agarose (1.5% w/v). Cells were incubated for 4 days to generate spheroids of 400 *μ*m in diameter. They were then treated with 2, 5, or 10 *μ*M of free raloxifene or SMA-Ral for 15 days. Media and treatment were renewed every three days. At the end of the treatment period, photographs were taken and cell viability of the tumor spheroids was assessed by an acid phosphatase assay as previously described [35]. Briefly, tumor spheroids were collected, washed in phosphate buffered saline, and incubated in the presence of acid phosphatase buffer (0.1 M sodium acetate, 0.1% Triton X-100, and p-nitrophenyl phosphate (2 mg/mL)) for 90 min at 37°C. The reaction was stopped with NaOH (1 N) and quantified at 405 nm on a microplate reader. The results are expressed as percentage of control. The three independent experiments were performed in sextuplicate.

#### 2.2.17. Statistics

Groups were compared using a one-way ANOVA. In all cases, the ANOVA was coupled with the Student-Newman-Keuls post hoc test. For all analyses, *P* < 0.05 was the minimal requirement for a statistically significant difference.

## 3. Results

### 3.1. Characterization of SMA-Ral

The poor solubility of raloxifene can be significantly improved by forming polymeric micelles from assembled amphiphilic SMA block copolymers. While free raloxifene is insoluble in water, the SMA-Ral micelles could be easily dissolved with an apparent solubility of 10.6 mg/mL (Table 1). The UV-spectrophotometry profile of the micellar drug was similar to that of free raloxifene. In the current study, we achieved loading of 20% as determined by the weight ratio of raloxifene over SMA. SMA-Ral had a mean micelle diameter of 65.34 ± 30.89 nm and a polydispersity index of 0.135 as measured by dynamic light scattering (Table 1). Nanomedicines with diameters greater than 7 nm evade renal filtration and urinary excretion [36]. The charge of SMA-Ral was near neutral with a zeta potential of −0.0165 mV (Table 1). Having a neutral zeta potential is helpful in decreasing the recognition of the micelle by the reticuloendothelial system (RES) composed of macrophages [37] and could prolong its presence in the circulation upon parenteral administration.

### 3.2. Release of Raloxifene from SMA-Ral

**Figure 1:**
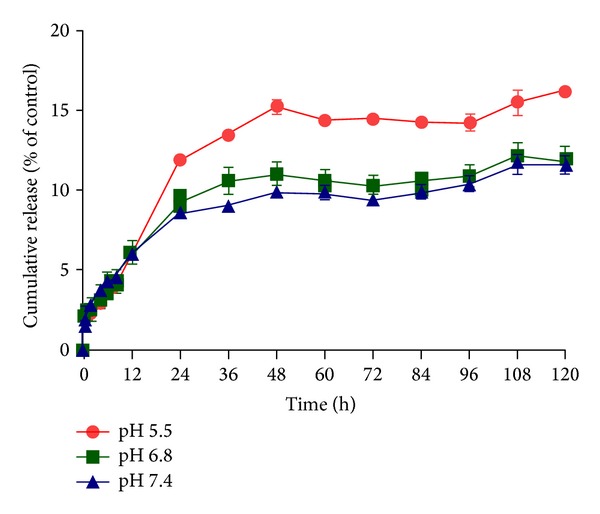
Release rate of raloxifene from SMA-Ral at pH 5.5, 6.8, and 7.4. The release of raloxifene was evaluated using dialysis method and compared to raloxifene present inside the dialysis bag at *t* = 0 h. The released was assessed over a period of 5 days. Data are expressed as mean ± SEM (*n* = 3) (*P* < 0.05 for pH 5.5 versus pH 6.8 and 7.4 from 24 to 120 h).

We compared the release rate of raloxifene at pH 5.5, 6.8 and 7.4 (Figure 1). At pH 7.4, similar to that of plasma, the cumulative release of raloxifene was 11.6% over 5 days of incubation. At pH 6.8, a comparable value to the surrounding of tumor tissue, the release of raloxifene was similar to that of pH 7.4 (11.9%). This release rate is optimal for a prolonged circulation time, protection from metabolizing enzymes, and ultimate tumor accumulation. The SMA-Ral had a faster release profile at pH 5.5, which corresponds to that found in the lumen of late endosomes with a cumulative release of 16.2% after 5 days of incubation (Figure 1). The stable micelles would thus benefit protecting raloxifene until it reaches tumor cells and then could be internalized and released through membrane hydrophobic partition, a unique intracellular release mechanism of SMA micellar system as described by Nakamura et al. [38].

### 3.3. Cytotoxicity of SMA-Ral

The cytotoxicity of SMA-Ral micelles was assessed *in vitro* over 72 h in CRPC cell lines, namely, PC3 and DU145 cells, and compared to the free drug. Treatment with SMA-Ral showed a higher cytotoxic effect in both cell lines compared to free raloxifene (Table 2). SMA (24 *μ*g/mL) and DMSO (0.05%) controls showed no cytotoxicity in both cell lines over the same period of treatment.

**Figure 2:**
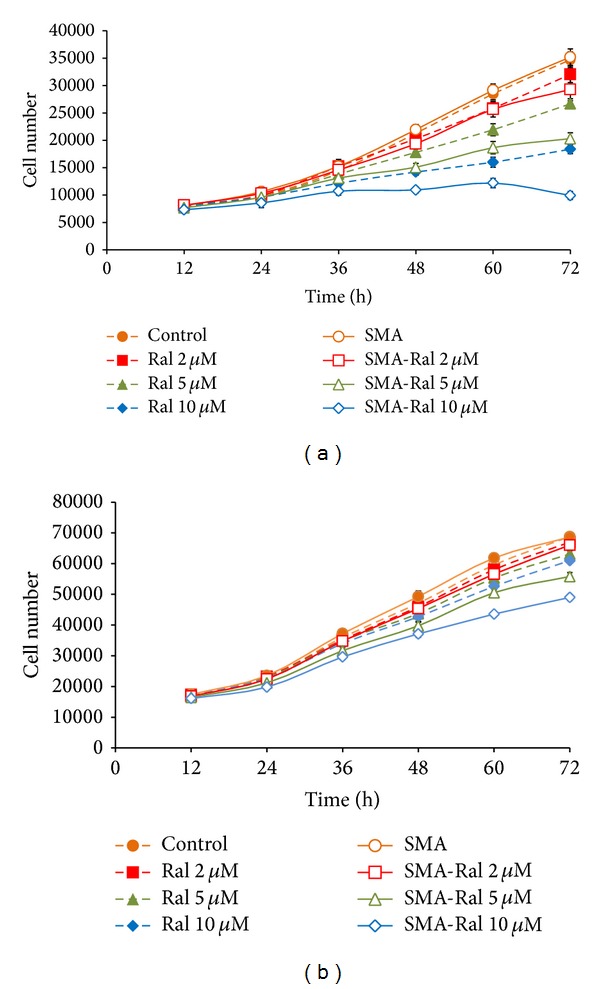
Comparison of the effect of various concentrations of raloxifene (Ral) and SMA-Ral (SMA-Ral) on the proliferation of PC3 (a) and DU145 (b) cells. Cells were treated over a period of 72 h with specific concentrations of raloxifene or SMA-Ral. At the indicated time point, cells were fixed and cell number was determined using the sulforhodamine B assay. Data are expressed as mean ± SEM (*n* = 3).

The effects of specific concentrations of free raloxifene and SMA-Ral were assessed over a time course of 72 h in PC3 and DU145 cells. PC3 cells were more sensitive to raloxifene than DU145 cells (Figure 2). In both cell lines, the SMA-Ral treatments were more potent than free raloxifene (Figure 2). The differences between the cell lines in their sensitivity to raloxifene may be dependent on their gene expression patterns, where PC3 cells express both ER*α* and ER*β* but DU145 cells only have ER*β* [39].

After 48 h, a significant difference in the cell numbers was observed between controls and SMA-Ral treatments at 5 *μ*M and 10 *μ*M in both PC3 and DU145 cells (Figure 2). Taken together, these data indicate that the effects of free raloxifene and SMA-Ral were cell specific and time dependent.

### 3.4. Effect of Raloxifene and SMA-Ral on DNA Synthesis and Cell Proliferation

**Figure 3:**
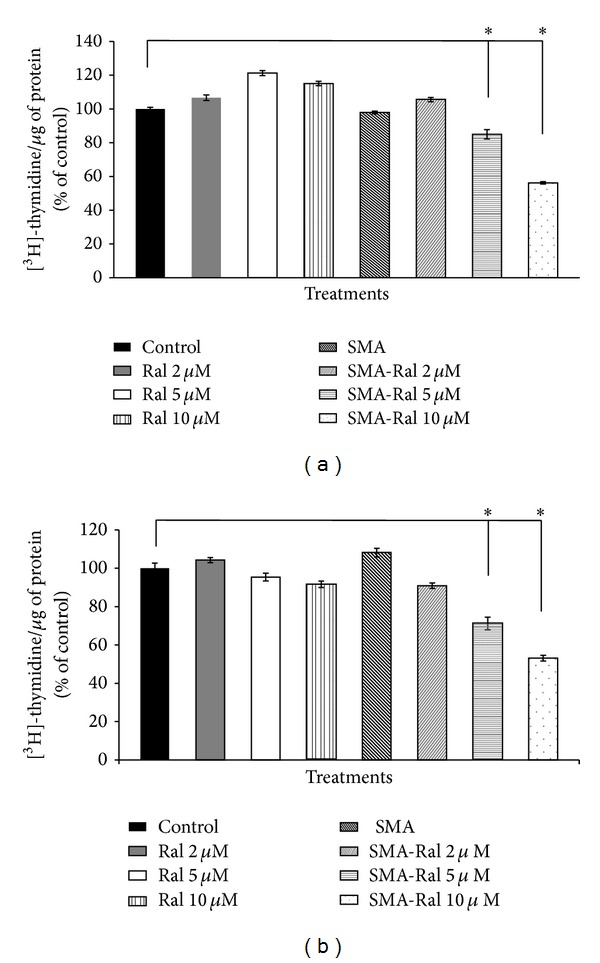
DNA synthesis following treatment of PC3 (a) and DU145 cells (b) with free raloxifene or SMA-Ral. Cells were treated with various concentrations of free raloxifene (Ral) or SMA-Ral for 48 h. DNA synthesis was evaluated by [^3^H] thymidine incorporation during the last 20 h of the treatment. Data are expressed as mean ± SEM (*n* = 3).

In order to determine the effect of raloxifene and SMA-Ral on DNA synthesis, PC3 and DU145 cells were treated for 48 h with specific concentrations of free raloxifene and SMA-Ral and [^3^H]-Thymidine incorporation was measured. Exposing PC3 and DU145 cells to free raloxifene (2–10 *μ*M) did not alter DNA synthesis when normalized to protein content (Figure 3). However, treatment with SMA-Ral significantly decreased DNA synthesis in both cell lines. At concentrations of 5 and 10 *μ*M, [^3^H]-Thymidine incorporation was decreased by 15 and 44%, respectively, in PC3 cells and by 29 and 47%, respectively, in DU145 cells (Figure 3).

**Figure 4:**
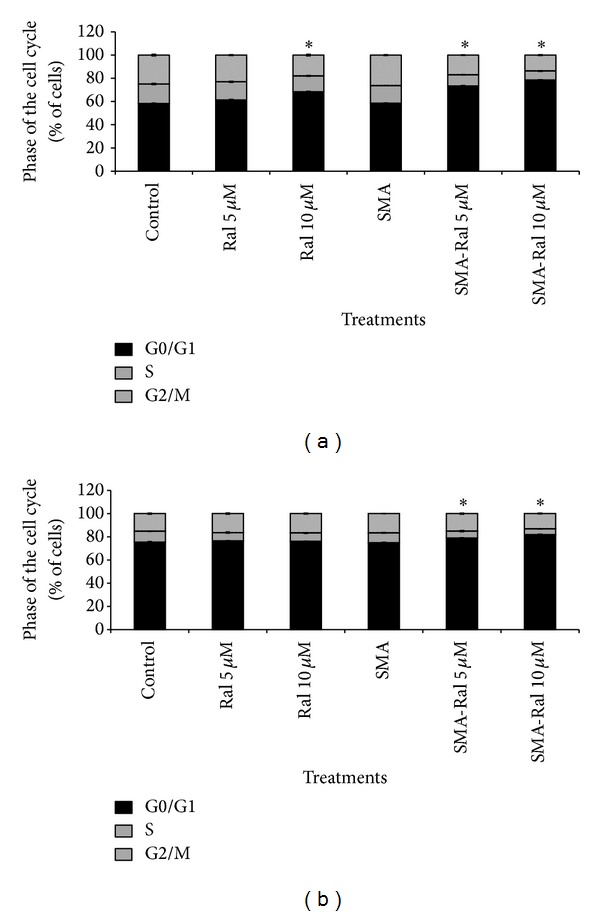
Effect of free raloxifene and SMA-Ral on cell cycle progression. PC3 (a) and DU145 cells (b) were treated for 48 h with either free raloxifene, SMA-Ral at 5 or 10 *μ*M, or controls (SMA or DMSO). Data are expressed as mean ± SEM (*n* = 3). *P < 0.05 compared to controls.

To determine whether SMA-Ral micelles were capable of inducing cell cycle arrest, flow cytometry was used on both PC and DU145 cell lines. As shown in Figure 4, the treatment of CRPC cells with SMA-Ral was associated with a higher number of cells in the G0/G1 phase of the cell cycle. In PC3 cells, free raloxifene did not affect cell cycle progression at concentrations below 10 *μ*M. At 10 *μ*M a small increase of cells in G0/G1 phase of the cell cycle was observed (10%) with a concomitant decrease of cells in S-phase (−3%) and G2/M-phase (−7%) (Figure 4(a)). However, treatment of PC3 cells with SMA-Ral 5 and 10 *μ*M potentiated G0/G1 arrest and increased the percentage of cells by 15 and 20% in G1/G0 phase with a concomitant reduction of S-phase (−5 and 7%) and G2M phase (−10 and 13%), respectively (Figure 4(a)). In DU145 cells, free raloxifene treatments did not affect the cell cycle progression at the concentrations here used (Figure 4(b)). SMA-Ral treatment slightly increased the number of cells in G0/G1 phase of the cell cycle by 4 and 7% for SMA-Ral concentration of 5 and 10 *μ*M, respectively (Figure 4(b)).

Overall, SMA-Ral treatment was more potent compared to free raloxifene in PC3 and DU145 cells. SMA-Ral reduced DNA synthesis as well as halted the progression of cells through the cell cycle. The effect of SMA-Ral was concentration dependent and more potent in PC3 cells compared to DU145 cells.

### 3.5. Effect of Raloxifene and SMA-Ral on Apoptosis and Necrosis

**Figure 5:**
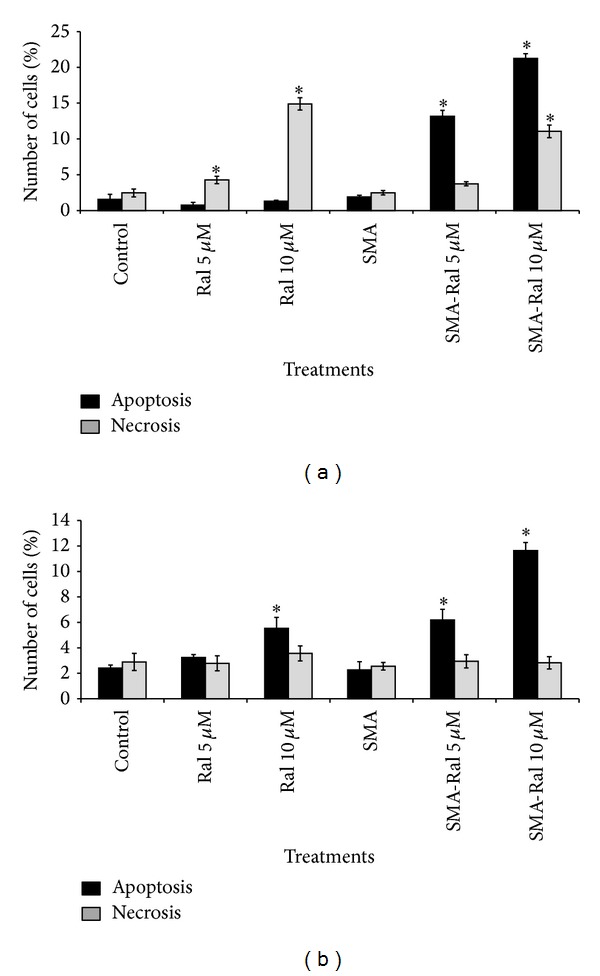
Effect of free raloxifene and SMA-Ral on apoptosis and necrosis. PC3 (a) and DU145 cells (b) were treated for 48 h with either free raloxifene (Ral), SMA-Ral at 5 or 10 *μ*M, or controls (SMA or DMSO). Data are expressed as mean ± SEM (*n* = 3). (*P < 0.05 compared to control).

We used flow cytometry to determine if free raloxifene or SMA-Ral treatments were able to induce apoptosis or necrosis. Apoptosis was measured using FITC-Annexin V which recognized the externalization of phosphatidylserine, a common characteristic of apoptotic cells. The proportion of necrotic cells was determined by propidium iodide. Treatment of PC3 cells with free raloxifene did not promote apoptosis but triggered a concentration-dependent necrosis, with 2-fold and 6-fold increase of necrotic cells with 5 and 10 *μ*M, respectively (Figure 5(a)). Interestingly, SMA-Ral treatment promotes apoptosis in PC3 cells, with 7- and 11-fold increase of apoptotic cells following treatment with 5 and 10 *μ*M, respectively (Figure 5(a)). In addition, cell necrosis was concentration dependent with 1.5- and 4-fold increase following 5 and 10 *μ*M raloxifene, respectively. The sensitivity of DU145 cells to treatment with raloxifene differed largely from the sensitivity of PC3 cells. Free raloxifene or SMA-Ral failed to trigger necrosis at 5 or 10 *μ*M. Free raloxifene increased apoptosis by 2-fold following 10 *μ*M treatment (Figure 5(b)) whereas SMA-Ral increased the occurrence of apoptosis by 2- and 6-fold following treatment with 5 and 10 *μ*M SMA-Ral (Figure 5(b)).

Overall, these data demonstrated that the mechanisms of inducing cell death differ between free raloxifene and SMA-Ral in PC3 cells. SMA-Ral induces apoptosis while free raloxifene elicits necrosis. DU145 cells are less sensitive to raloxifene treatment but SMA-Ral potentiates apoptosis. The difference in sensitivity between PC3 and DU145 cells may also involve different signaling mechanisms leading to different internalization and subcellular localization inherent to the endocytic process characteristic of macromolecular cell uptake.

### 3.6. Effect of SMA-Ral on the Expression of Protein Involved in Proliferation and Protein Synthesis

**Figure 6:**
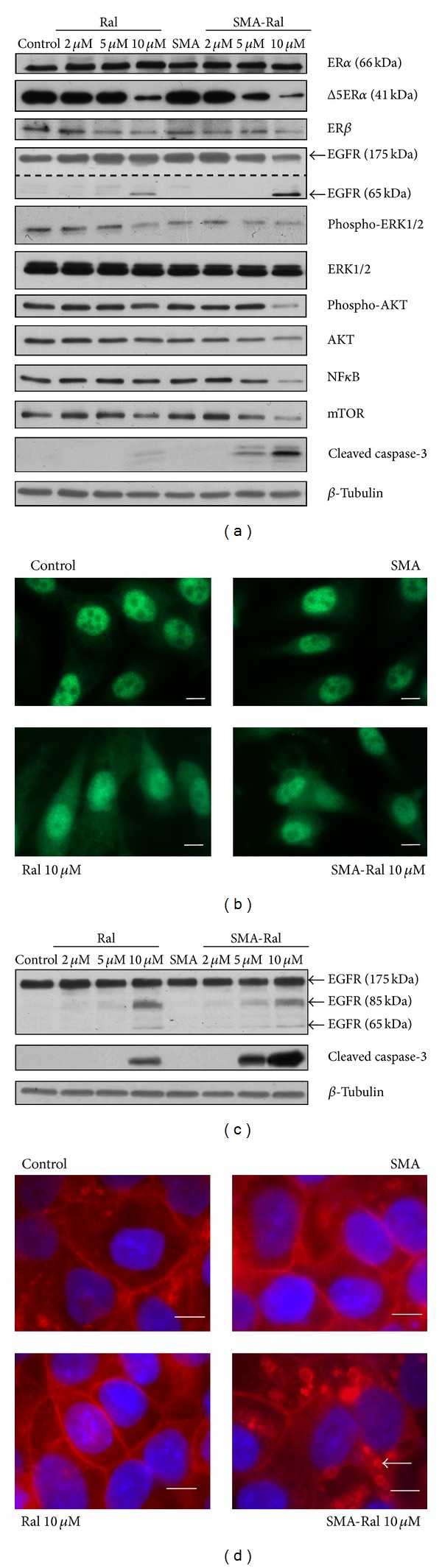
Effect of free raloxifene and SMA-Ral on ER*α* and ER*β* protein expressions and proteins involved in cell cycle progression and protein synthesis. Western blot of proteins following treatment with free raloxifene or SMA-Ral at 2, 5, and 10 *μ*M for 48 h in PC3 cells (a). Immunocytochemistry of ER*β* following treatment with free raloxifene or SMA-Ral 10 *μ*M (b). Western blot of EGFR in DU145 cells treated with free raloxifene or SMA-Ral at 2, 5, and 10 *μ*M for 48 h (c). Localization of EGFR in DU145 cells treated with free raloxifene or SMA-Ral 10 *μ*M for 48 h (d).

To determine the mechanism for the higher sensitivity of PC3 cells to SMA-Ral treatment, we examined the effect of free raloxifene and SMA-Ral on the expression of several proteins involved in cell proliferation and protein synthesis. As shown in Figure 6, treatment with free raloxifene or SMA-Ral did not affect the expressions of ER*α* (66 kDa). However, the expression of a Δ5ER*α*, a splice variant of ER*α*, was decreased by 70% following free raloxifene (10 *μ*M) treatment. SMA-Ral further potentiates the decreased expression of Δ5ER*α* and that at a lower concentration. Δ5ER*α* expression was decreased by 60 and 90% following 5 and 10 *μ*M SMA-Ral treatment. The expression of ER*β* was not modified by raloxifene; however, its nuclear localization was decreased both by raloxifene and SMA-Ral (Figure 6(b)), suggesting a decrease of ER*β* binding to the estrogen response element (ERE). Crosstalk has been documented between ER*α*, ER*β*, and other signaling proteins involved in cell proliferation and protein synthesis such as the epidermal growth factor receptor (EGFR), mitogen activated protein kinase (MAPK, ERK1/2), or serine/threonine kinase (AKT). We examined the effect of free raloxifene and SMA-Ral on the expression of these proteins. Treatment with free raloxifene from 2 to 10 *μ*M had no effect on EGFR protein expression; however, 10 *μ*M of free raloxifene promoted the appearance of an EGFR fragment of approximately 65 kDa. Treatment with 5 and 10 *μ*M SMA-Ral resulted in a decrease in EGFR expression by 27 and 36%, respectively. The appearance of a truncated form of EGFR (65 kDa) was also observed with SMA-Ral 10 *μ*M and this concentration also decreased ERK1/2 as well as AKT phosphorylation and expression, both signaling pathways involved in cell proliferation and inhibition of apoptosis, along with other proteins involved in protein synthesis such as NF*κ*B and mTOR. In addition, treatment with 5 and 10 *μ*M SMA-Ral promoted the activation of caspase-3, a marker for apoptosis mediated cell death while free raloxifene treatment 10 *μ*M only showed a faint activation of caspase-3 (Figure 6(a)).

In DU145 cells, mechanisms implicated in the reduction of cell proliferation and increased apoptosis are mediated through different pathways as the proteins modified by SMA-Ral treatment in PC3 were not affected in DU145 cells. In DU145 cells, raloxifene did not affect EGFR expression; however, with free raloxifene (10 *μ*M) and SMA-Ral (5 and 10 *μ*M), cleaved forms appeared with apparent molecular weights of 85 kDa and 65 kDa (Figure 6(c)). In addition, treatment with SMA-Ral 10 *μ*M specifically promoted endocytosis of EGFR (Figure 6(d)).

Overall, the effect of SMA-Ral on downstream signaling effectors appeared dependent on cell type. SMA-Ral decreased the expression and activation of proteins involved in the regulation of cell proliferation and protein synthesis in PC3 cells. In DU145 cells, cells are less sensitive to SMA-Ral treatment and the mechanisms may be mediated through a decreased expression of the EGFR at the membrane and subsequent downstream effectors.

### 3.7. Effect of Raloxifene and SMA-Ral on the Integrity of PC3 Tumor Spheroids

**Figure 7:**
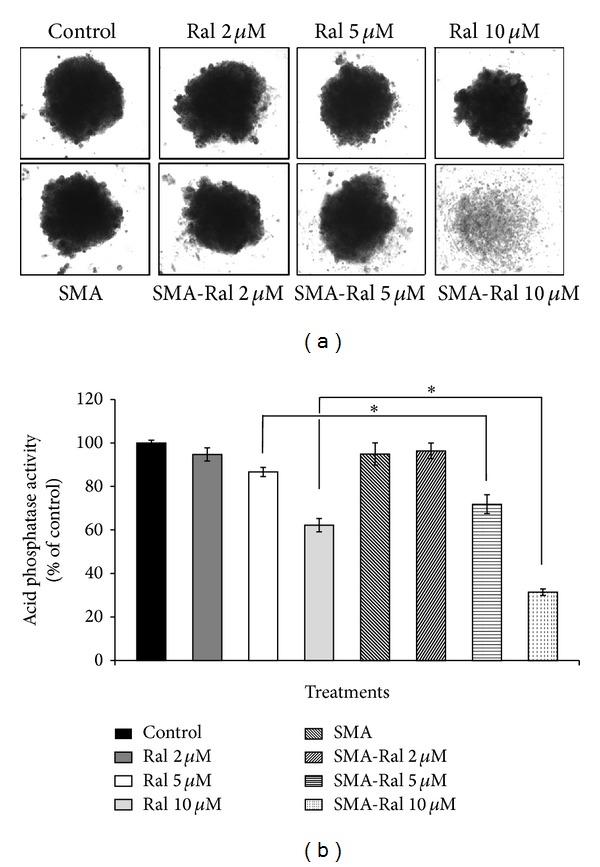
Morphologies and viability of PC3 tumor spheroids after 15 days of treatment with free raloxifene or SMA-Ral. Representative pictures of tumor spheroids were taken following treatment with free raloxifene or SMA-Ral at 2, 5, and 10 *μ*M or controls (DMSO or SMA) (a). Tumor spheroid viability was measured by the activity of acid phosphatase (b). *P < 0.05.

We compared the efficacy of free raloxifene and SMA-Ral using PC3 tumor spheroids since, as previously reported by Friedrich et al. [35], growth of DU145 tumor spheroids failed. PC3 tumor spheroids were treated over a period of 15 days with either free raloxifene or SMA-Ral at 2, 5 and 10 *μ*M. As shown in Figure 7, morphologies of PC3 tumor spheroids was not modified upon free raloxifene treatment with 2 or 5 *μ*M (Figure 7(a)). Concentrations of free raloxifene up to 10 *μ*M decreased the spheroid volume by 16% and by 38% the activity of acid phosphatase, a marker of cell viability (Figure 7(b)). In contrast, treatment with SMA-Ral 5 *μ*M reduced the spheroid volume by 11% and decreased acid phosphatase activity by 29% compared to control or SMA and 15% compared to free raloxifene 5 *μ*M. The effect of SMA-Ral on the integrity of tumor spheroids was concentration dependent; SMA-Ral (10 *μ*M) abolished their spherical morphology resulting in cellular aggregates without defined structure (Figure 7(a)). This was accompanied by a decrease in cell viability of 69% compared to control or SMA and 30% compared to free raloxifene 10 *μ*M (Figure 7(b)). These results provided further evidence of the potency of SMA-Ral.

### 3.8. Effect of Free Raloxifene and SMA-Ral on PC3 Cell Migration and Invasion and MMP-9 Secretion

**Figure 8:**
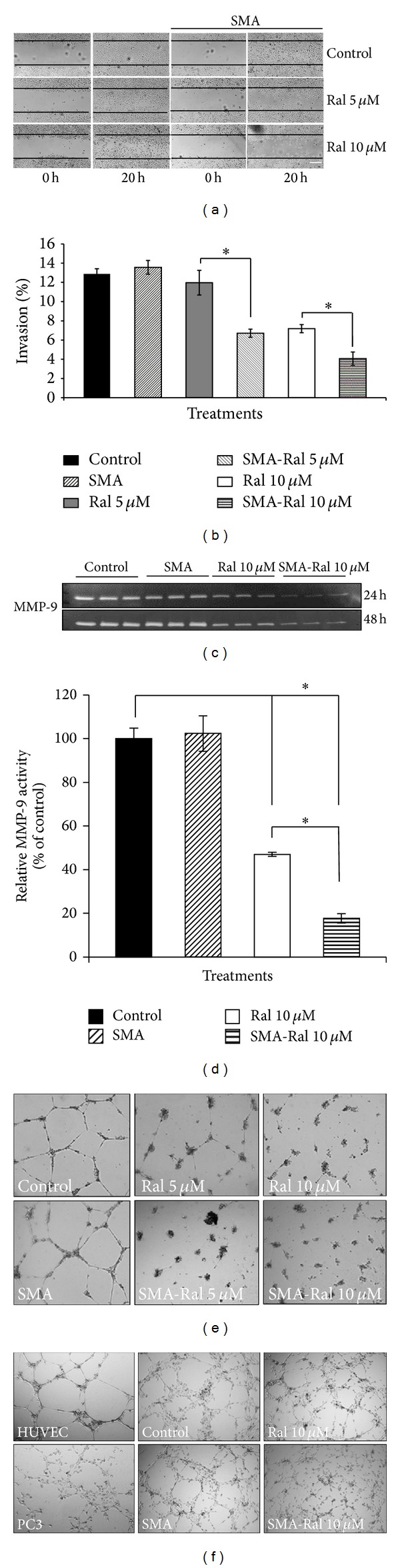
Effect of free raloxifene or SMA-Ral treatment on cell migration and invasion. PC3 monolayer of cells was scratched and treated with either free raloxifene or SMA-Ral at 5 or 10 *μ*M or controls (DMSO or SMA) and incubated for 20 h. Representative pictures were taken at *t* = 0 h and at 20 h (a). For cell invasion, PC3 cells were treated with either free raloxifene or SMA-Ral at indicated concentrations. After 20 h-the cells migrating to the lower surface were fixed and stained with Diff Quick. Bars represent the mean ± SEM of three independent experiments (b). Conditioned mediums were collected from cultures following 24 and 48 h and analyzed by gelatin zymography (c). Bars indicate the relative MMP-9 activity in the conditioned media and represent the mean ± SEM of three independent experiments (d). Effects of free raloxifene and SMA-Ral on the formation of capillary-like structures by HUVEC. Cells were treated with either free raloxifene or SMA-Ral at 5 or 10 *μ*M or controls (DMSO or SMA) for 24 h. Representative pictures were taken (e). PC3 cells cocultured with endothelial cells were treated with either free raloxifene or SMA-Ral at 10 *μ*M or controls (DMSO or SMA) for 24 h. Representative pictures were taken (f). *P < 0.05.

We have demonstrated that SMA-Ral decreased cell viability, proliferation and affected the integrity and viability of tumor spheroids. Next, we tested the effect of free raloxifene and SMA-Ral on the migration, and invasion of PC3 cells. Migration was determined using a scratch assay. Treatment with free raloxifene 5 and 10 *μ*M, decreased migration of PC3 cells in a concentration-dependent manner (Figure 8(a)). Moreover, SMA-Ral reduced migration by 50 and 90% when treated with 5 and 10 *μ*M, respectively (Figure 8(a)). SMA-Ral also elicited a concentration dependent reduction of matrigel invasion. While 5 *μ*M free raloxifene had no effect, treatment at 10 *μ*M decreased invasion by 1.6-fold (Figure 8(b)). In contrast, SMA-Ral reduced cell invasion by 1.9- and 3-fold following treatment with 5 and 10 *μ*M (Figure 8(b)). Matrix metalloproteinase (MMP) secretion has been implicated with cancer invasiveness and tumor progression [40]. Analysis of the secretion level of MMP-9 in the conditioned media after 24 and 48 h by gelatin zymography showed that free raloxifene and SMA-Ral 10 *μ*M decreased MMP-9 secretion after 24 and 48 h incubation. Additionally, after 48 h incubation, MMP-9 secretion was decreased by 53% and 83% by 10 *μ*M of free raloxifene and SMA-Ral, respectively (Figures 8(c)-8(d)). We also compared the effect of raloxifene and SMA-Ral on endothelial tube formation using HUVEC cells. While raloxifene 10 *μ*M decreased endothelial tube-like formation, SMA-Ral 5 *μ*M abolished tube-forming capability (Figure 8(e)). In addition, the coculture of PC3 cells and endothelial cells on a basement membrane matrix surface promoted the interaction between the two cell lines and the formation of tubule (Figure 8(f)). These interactions were decreased efficiently by the treatment with SMA-Ral 10 *μ*M (Figure 8(f)) suggesting that the treatment with SMA-Ral will decrease neoangiogenesis in the tumor.

## 4. Discussion

In the present study, we report a new raloxifene formulation with higher cytotoxicity against CRPC cell lines compared to free raloxifene. The encapsulation of raloxifene into SMA micelles resulted in reduced CRPC cell proliferation, promoted cell death, impaired tumor spheroid formation, decreased interaction with endothelial cells, reduced cell migration and invasion more effectively than the free drug.

Free raloxifene transport into the cell depends on active and saturable carriers belonging to the organic anion transporting polypeptide family (OATP). Two well-studied members of this family, OATP1B1 and OATP1B3, have been implicated in the internalization of raloxifene [41]. Interestingly, in prostate cancer, the expression of OATP1B3 has been shown to be upregulated [42]. SMA-Ral crosses the plasma membrane independently of carriers, delivering raloxifene to the cytoplasm away from the plasma membrane and the efflux drug transporters such as P-glycoprotein (Pgp), multidrug resistance-related protein (MRP), and OATP are also involved in the excretion of raloxifene [43, 44]. MRP was also found to be expressed in PC3 and DU145 cells [45].

Many studies have demonstrated that the cellular uptake of nanoparticles involves either clathrin-mediated or caveolae-mediated endocytosis [46, 47]. Treatment of PC3 cells with 10 *μ*M SMA-Ral triggered the formation of caveolin-1 vesicles after 48 h of incubation but did not promote the formation of clathrin vesicles (data not shown). Endocytosis is a multistep process that leads to lysosome formation which promotes the degradation of nanoparticles due to the acidic environment (lumen pH 5.5), releasing their content into the cytosol [48]. Once raloxifene is delivered by endocytosis into the cytosol, it modulates signaling pathways that are different from those mediated by the free drug resulting in the decrease of cell proliferation and/or the increase of cell death.

We have also shown that SMA-Ral treatment reduced DNA synthesis (Figure 3) and cell proliferation by promoting the accumulation of cells in the G1 phase of the cell cycle for PC3 and DU145 cells (Figure 4). In addition, we showed that free raloxifene and SMA-Ral induced cell death potentially through different mechanisms (Figure 5). In PC3 cells, while free raloxifene treatment mediated cell death through a necrotic process, the treatment with SMA-Ral implicated mainly apoptosis (Figure 5(a)). Previous studies have shown that SERMs can induce cell death through multiple mechanisms [49]; in addition, treatment with high concentration of SERM may also promote cell death independently of the activation of caspase [50]. As shown in Figure 6, caspase-3 is activated following treatment with SMA-Ral 10 *μ*M. In DU145, free raloxifene and SMA-Ral treatments induced apoptosis; however, the SMA-Ral treatment appeared more potent compared to free raloxifene. Previous studies using PC3 and DU145 cells have demonstrated cytotoxicity of free raloxifene at a low concentration (1 *μ*M) in cells incubated in steroids-stripped media [51]. These data suggested that raloxifene mediated cell death is dependent on the presence of steroids as higher concentrations of raloxifene are required to readily elicit potent cytotoxicity. The higher efficacy of the SMA-Ral might be explained by the release of the drug in the cytoplasm and direct targeting of ER*α* and ER*β*.

SMA-Ral cytotoxicity appeared more potent against PC3 cells that express both ER*α* and ER*β*, compared to only ER*β* in DU145 cells. Previous studies have hypothesized that the clinical benefit of SERMs for the treatment of CRPC relies on targeting ER*α* [52]. Moreover, raloxifene has a 17-fold higher affinity for ER*α* compared to ER*β* [53]. An additional parameter to consider is that the cellular localization of ER*α* and ER*β* differs where ER*α* is both cytoplasmic and nuclear in PC3 cells [54], while ER*β* is mainly localized in the nucleus of cells (Figure 6). The endocytosis process of SMA-Ral and the release of raloxifene in the cytoplasm may promote its interaction with the ER*α*. However, ER*β* is significantly expressed in human prostate cancer cells (including PC3 and DU145) and its potential as a raloxifene target in CRPC remains to be determined. Therefore, we cannot exclude a synergy between ER*α* and ER*β* to promote strong raloxifene-induced cytotoxicity.

To gain better understanding of the signaling mechanisms behind the effect of SMA-Ral in PC3 cells, we examined the expression of ER*α* and ER*β*. SMA-Ral treatment did not significantly alter the expression of full length ER*α*. However, ER*α*Δ5 expression, one of the splice variant of ER*α* characterized by the deletion of the ligand binding domain (exon 5, AF2 domain), was decreased in PC3 cells (Figure 6). Conflicting reports have associated the specific increased ER*α*Δ5 expression to the stimulation of gene expression in tumors [55]. SMA-Ral treatment decreased the expression of ER*α*Δ5 at concentrations as low as 5 *μ*M compared to 10 *μ*M with free raloxifene (Figure 6). ER*β* protein level was slightly decreased by free raloxifene or SMA-Ral treatment. ER*β* protein is essentially localized in the nucleus but treatment by both free raloxifene and SMA-Ral causes ER*β* to accumulate in the cytoplasm (Figure 6), suggesting that raloxifene might reduce the expression of genes normally targeted by ER*β* binding to their promoter. Analyzing the expression and/or activation of downstream signaling pathways in PC3 cells revealed that SMA-Ral treatment decreased activation or lowered expression of proteins involved in the proliferation or inhibition of apoptosis such AKT and ERK1/2 as well as in protein synthesis such as NF*κ*B and mTOR at concentrations equivalent to 5 *μ*M raloxifene. These data suggest the specificity and potential of SMA-Ral treatment for the treatment of CRPC expressing ER*α* and ER*β*.

In contrast, the mechanisms by which SMA-Ral treatment caused cytotoxicity to DU145 cells appeared to be mediated through different pathways all together, as none of the proteins examined in PC3 cells were affected by raloxifene. However, treatment with free raloxifene and SMA-Ral promoted the appearance of truncated EGFR protein, the role of which needs to be characterized in further studies. In addition, SMA-Ral induced the endocytosis of EGFR and its localization in cytoplasmic vesicles as shown by immunofluorescence analysis (Figure 6(d)). SMA-Ral treatment also triggered the formation of cytoplasmic vesicles containing EGFR in PC3 cells (data not shown). EGFR is highly expressed in DU145 cells compared to PC3 cells [56] and has been demonstrated to contribute to the proliferation of androgen independent prostate cancer cells [57]. Generation of the truncated form of EGFR receptors as well as its delocalization into cytoplasmic vesicle may delay proliferation and induce cytotoxicity in DU145 cells.

In addition, using the PC3 tumor spheroid model, we demonstrated that SMA-Ral destroyed the integrity and reduced the cell viability of the spheroids (Figure 7). Tumor spheroids are an advantageous *in vitro* model used to mimic specific characteristics of tumor development and the complex cellular interactions observed *in vivo* [58]. Tumor spheroids are also used to more accurately predict drug efficacy prior to examination *in vivo*. The potency of SMA-Ral against CRPC prostate tumor spheroids suggests strong potential and the need to further assess its value in preclinical animal models. Furthermore, SMA-Ral also decreased cell migration, cell invasion, and MMP-9 protein excretion in conditioned media of PC3 cells. The activity of MMPs has been repeatedly associated with the metastatic potential of tumor cells [59]. These data suggest the potency of SMA-Ral for the prevention of the appearance of metastasis *in vivo*. In addition, the interaction of PC3 cells with endothelial cells was significantly altered by the treatment with SMA-Ral which abolishes the tubule formation suggesting a potent antiangiogenic effect of SMA-Ral (Figure 8(e)).

Raloxifene is characterized by low bioavailability (approximately 2%) due to extensive metabolism [60] which essentially occurs via glucuronidation catalyzed by UDP-glucuronosyltransferases (UGTs) present in the liver [60]. Raloxifene encapsulation into the SMA micelles is plausibly protected from the liver metabolizing enzymes and could hypothetically improve its plasma level. These water soluble nanoparticles should promote the accumulation of raloxifene at the tumor site while protecting the drug from metabolic deactivation. In addition, the stability of SMA-Ral, demonstrated by the low release rate over 5 days (Figure 1), should increase its internalization by the prostate cancer cells. Further *in vivo* testing of these assumptions is currently being pursued in our laboratory.

The passive accumulation of nanoparticles at tumor sites has been demonstrated by Matsumura and Maeda [61] who established the concept of the enhanced permeability and retention (EPR) effect. The EPR effect is associated with irregular blood vessel morphology inherent to tumor tissues and characterized by large fenestration between the endothelial cells. The EPR effect promotes the accumulation of nanomedicine at tumor sites by passive targeting while its particle size prevents extravasation from normal vessels. Passive targeting can prolong retention of a drug in the tumor interstitium over days to weeks [62]. In addition, the reduced clearance of SMA-Ral due to its size and charge will contribute to its accumulation at the tumor site as well as in distant metastatic secondary tumors. A nanoparticle diameter larger than 7 nm will escape the renal filtration through the glomerular slits and remain in the systemic circulation for a longer period [63–65]. In the systemic circulation, the micellar charge dictates the molecules interaction with blood components, vascular endothelium, or the reticuloendothelial system (RES). A neutral zeta potential as measured for SMA-Ral is expected to decrease recognition of the micelle by the components of the RES and prolong its circulation [66]. The stability of the SMA-Ral as observed by the low release rate (only 12% release after 5 days incubation at physiological pH 7.4) (Figure 1) will extend its circulation time and may promote drug localization at the tumor site.

## 5. Conclusion

Together, the data obtained in this study demonstrated the advantages of encapsulating raloxifene into SMA and its cytotoxic potency in two CRPC cell lines differing in the level of ER*α* and ER*β* expression. Compared to free drug, SMA-Ral more effectively inhibits cell cycle progression, increases apoptosis, and alters the integrity of tumor spheroid models. SMA-Ral treatment decreased migration and invasion of a CRPC cells. The micellar system could possibly have different mechanisms of action compared to free drug. This hypothesis is supported by the distinct pattern of expression and localization of estrogen receptors, EGFR, and downstream signaling of cell proliferation and survival. This new formulation could potentially confer a superior efficacy and pharmacokinetic profile to raloxifene and thus warrants further examination *in vivo*.

## Figures and Tables

**Table 1:** SMA-Ral micelles characterization.

SMA-Ral	Measurement
Drug loading efficiency (%)	87
Drug loading (% w/w)	20
Mean micelle diameter (nm)	65.34 ± 30.89
Polydispersity index	0.135 ± 0.021
Zeta potential (mV)	−0.0165 ± 4.59
Solubility (mg/mL)	10.6

**Table 2:** IC_50_ values for raloxifene free drug and SMA-Ral in HRPC cells lines.

Cells	SMA-Ral (M)	Free drug (M)
PC3	7.75*E* − 06	1.03*E* − 05
DU145	1.03*E* − 05	1.46*E* − 05
